# Calcaneal quantitative ultrasound parameters strongly correlate with DXA measurements in patients with acromegaly

**DOI:** 10.1007/s11102-026-01696-4

**Published:** 2026-05-18

**Authors:** Claudia Campana, Andrea Casabella, Anna Arecco, Maurizio Cutolo, Serena Tonelli, Elvis Hysa, Lara Vera, Francesca Bertaina, Jessica Amarù, Diego Ferone, Sabrina Paolino, Federico Gatto

**Affiliations:** 1 Department of Internal Medicine and Medical Specialties (DIMI), Endocrinology Unit, School of Medical and Pharmaceutical Sciences, University of Genoa, Genoa, Italy; 2 IRCCS Azienda Ospedaliera Metropolitana, Genoa, Italy; 3 Laboratory of Experimental Rheumatology and Academic Division of Clinical Rheumatology, Department of Internal Medicine and Specialties, University of Genoa, Genoa, Italy; 4 Department of Experimental Medicine (DIMES), University of Genoa, Genoa, Italy; 5 Endocrinology Unit, IRCCS Azienda Ospedaliera Metropolitana, Genoa, Italy; 6 Endocrinology Unit, Department of Internal Medicine and Medical Specialties (DIMI), University of Genoa, Largo Rosanna Benzi, 10, Genoa, 16132 Italy

**Keywords:** Bone mineral density, Osteopenia, Osteoporosis, GH-secreting pituitary adenoma, Acromegaly

## Abstract

**Purpose:**

Patients with acromegaly have increased skeletal fragility. To date, dual-energy X-ray absorptiometry (DXA) and vertebral morphometry are the recommended examinations to evaluate bone health in these patients, despite known limitation. Calcaneal quantitative ultrasound (cQUS) is a less expensive and less invasive method to evaluate bone status compared to DXA. We aimed to investigate bone status by cQUS in a cohort of patients with acromegaly and to evaluate its ability to detect bone impairment.

**Methods:**

Observational cohort study including 56 patients with acromegaly (acromegaly group, AG) and 59 healthy subjects matched for age, sex and BMI (control group, CG). DXA and cQUS assessment were performed in both AG and CG; detailed clinical data were collected for the AG.

**Results:**

All cQUS parameters showed a good correlation with the DXA-derived T-score values (p<0.001) in both AG and CG. The cQUS-derived T-score was not significantly different in the AG compared to the CG (AG: 0.40 [IQR -1.60 to 1.50]; CG: -1,30 [IQR -1.65 to 0.95], p= 0.068). The cQUS-derived T-score showed a good discriminatory ability for the presence of osteopenia in the AG (AUC 0.833 [CI 0.721-0.945]). This discriminatory ability of the cQUS-derived T-score was similar between the AG and the CG (p=0.325).

**Conclusions:**

cQUS parameters showed a good correlation with DXA evaluation in patients with acromegaly, comparable to the observation in the general population. cQUS-derived T-score can accurately discriminate osteopenia in patients with acromegaly.

**Supplementary Information:**

The online version contains supplementary material available at 10.1007/s11102-026-01696-4.

## Introduction

Acromegaly is a rare systemic endocrine disease, caused in the vast majority of cases by a growth hormone (GH)-secreting pituitary adenoma. The GH excess and the subsequent increased secretion of insulin-like growth factor 1 (IGF-1) by the liver, contribute to multiple systemic comorbidities and to a reduced quality of life [1, 2]. GH and IGF-1 are both directly involved in bone metabolism, and their long-lasting excess results in increased bone turnover and deterioration. Indeed, acromegaly patients have increased skeletal fragility and a higher risk of vertebral fractures compared to the general population [3–5]. Moreover, other factors such as hypogonadism and the presence of diabetes mellitus (DM), which represent frequent comorbidities of acromegaly, can further increase fracture risk, together with the presence of a suboptimal disease control [6, 7]. Data concerning bone mineral density (BMD) assessed using dual-energy X-ray absorptiometry (DXA) in patients with acromegaly are conflicting, and the association between BMD values and the incidence of vertebral fractures is debated [6]. However, the last Consensus Statement on the management of acromegaly comorbidities recommends to perform a DXA evaluation every 2 years and a vertebral morphometry annually to assess the presence of vertebral fracture [3]. In recent years, several methods have been investigated in order to improve the assessment of bone quality in patients with acromegaly. Among these, the trabecular bone score (TBS), obtained from DXA scans, has been demonstrated to provide additional information on bone microarchitecture; however, to date, it is mostly used in research setting [8].

The calcaneal quantitative ultrasound (cQUS) is a less expensive and more accessible method compared to DXA, and it has been evaluated as a screening evaluation both in post-menopausal women and in different conditions associated with bone fragility (e.g., chronic obstructive pulmonary disease, type 2 diabetes mellitus [T2DM], and prostate cancer) [9–12].

Few studies, with limited sample size, have investigated the cQUS in the setting of acromegaly [13, 14]. These studies were primarily conducted on patients with active acromegaly, and the reported treatments were limited to surgery and/or radiotherapy.

In our study we aimed to investigate the bone status using cQUS in a cohort of patients with acromegaly and to compare the observed results with those obtained from the standard DXA and TBS evaluations. Moreover, we aimed to investigate the ability of cQUS to detect bone impairment in patients with acromegaly.

## Subjects and methods

Observational cohort study involving patients with acromegaly with active follow-up at a single tertiary center for pituitary diseases (Endocrinology Unit, IRCCS Azienda Ospedaliera Metropolitana, Genoa, Italy) between January 2023 and January 2025.

Diagnosis of acromegaly was made according to current guidelines, as previously described [15].

Inclusion criteria were: age > 18 years and the signed informed consent to perform a DXA and cQUS evaluation. Pregnancy status was excluded for women of childbearing age. A total of 56 patients with acromegaly (acromegaly group, AG) were included in the study.

Detailed clinical data were collected for all patients. In particular, we collected data related to treatment modalities for acromegaly, disease control and pituitary function, as well as the presence of concomitant comorbidities that impact bone health [16].

Hypogonadism was defined as irregular or absent menstrual cycles in women and a total testosterone level below 264 ng/mL in men with associated symptoms [17].

Fifty-nine healthy subjects matched for age, sex and BMI were enrolled as a control group (CG). Healthy subjects were selected among volunteers with no known clinical nor family history of osteoporosis or other bone-related diseases. In more detail, subjects with diseases or clinical conditions possibly leading to osteoporosis (hyperthyroidism/thyrotoxicosis, hypercortisolism, primary or secondary hyperparathyroidism, chronic renal failure, malabsorption, bedridden), as well as current or previous therapies that can impact bone structure (glucocorticoids as anti-inflammatory/immunosuppressive therapy, GnRH analogues, immunosuppressive drugs, antiretrovirals, anticoagulants, anticonvulsants, pioglitazone) were excluded from the study. The presence of uncontrolled DM (glycated haemoglobin [HbA1c] > 7.5%), active malignant neoplasms, and previous traumas represented additional exclusion criteria.

The study was conducted in accordance with the recommendations of the Declaration of Helsinki, and all patients gave written consent to use clinical data for research purposes. The study received approval from the Ethical Committee of Liguria Region (IRB number 245/2023).

### Laboratory assessment

The following hormonal and biochemical parameters were investigated in patients with acromegaly within three months of the radiological bone assessment: fasting plasma glucose, HbA1c, thyroid stimulating hormone (TSH), free thyroxine (fT4), prolactin, total testosterone (men), estradiol (pre-menopausal women), sex-hormone binding globulin, albumin, morning plasma cortisol, parathyroid hormone (PTH), 25-hydroxyvitamin D [25(OH) vitamin D], electrolytes, creatinine, bone specific alkaline phosphatase (BSAP) and C-terminal telopeptide (CTX).

Disease control was evaluated assessing IGF-1 values, measured with a chemiluminescent immunometric assay (Immulite 2000, Siemens Healthcare Diagnostics Products), calibrated to the WHO 87/518 IS, as previously described [18]. All the above-mentioned parameters were determined by the routine automatic methods in use at the Medicine Laboratory of our Institution (IRCCS Azienda Ospedaliera Metropolitana, Genova, Italy).

### Bone assessment

All patients with acromegaly and healthy controls underwent bone densitometry [DXA Lunar full-Prodigy (GE Lunar, Madison, WI, USA)] and quantitative BMD analysis. BMD of the lumbar spine (L1–L4), femoral neck and total hip was expressed in g/cm². Based on DXA evaluation, subjects were classified as having normal BMD and, in case of bone impairment, as having osteopenia (T-score = − 1.0 to − 2.4 SD in at least one of the considered sites) or osteoporosis (T-score ≤ − 2.5 SD in at least one of the considered sites) [19]. Body weight and height were also recorded, and body mass index (BMI) was calculated. Bone status was also qualitatively assessed using trabecular bone score (TBS) analysis [iNsight Medimaps Panel (GE Healthcare Needham, MA, USA, software version 3.0.0.15)], which was calculated at each spinal DXA examination. We considered TBS ≥ 1.350 as normal, TBS values ​​between 1.200 and 1.350 as consistent with partially degraded microarchitecture, while TBS values ​​≤ 1.200 as degraded microarchitecture [20]. The T-score modified by TBS was then calculated as previously described [21].

Calcaneus quantitative ultrasound (cQUS) was performed using an ultrasound scan (Osteosys BeeTLe, Caresmed) according to a standardized protocol. Trained staff ensured that participants were able to perform measurements on both heels, except for heels with open wounds, injuries, or metal implants. Daily quality control was performed using a phantom according to the manufacturer’s guidelines. The device measured the speed of sound (SOS) and broadband ultrasound attenuation (BUA), which are indicators of bone quality. In detail, SOS is mainly influenced by bone density and elasticity and reflects both quantity and quality of the bone. Higher SOS values generally reflect higher bone density and better elastic properties. Similarly, BUA is related to bone mass and architecture, with higher values generally reflecting a higher bone density and a more favourable bone architecture [22, 23]. If there was no history of fractures or other relevant foot disease, the left foot was examined (otherwise the right foot was measured). The cQUS bone quality index (BQI) was calculated by the instrument software, and a cQUS T-score was derived from it [24]. The coefficient of variation was 6.5% for BUA, 0.2% for SOS, and 2.8% for BQI.

### Statistical analysis

R version 4.4.0 was used for statistical analyses and to draw figures. Quantitative data are presented as median and interquartile range (IQR), while categorical variables are presented as frequencies and percentages. Between group comparisons were analysed by the Kruskal–Wallis test. Correlations were performed using Spearman’s rho correlation coefficient for ranks. The two-sided Chi-square test was used to evaluate differences in cross-tables. To evaluate the predictors of bone status (in particular DXA-derived T-score and TBS values) in the AG we performed a multivariable linear regression analysis and the stepwise selection based on the Akaike Information Criterion (AIC) was applied.

We used the Receiver Operating Characteristic (ROC) curve and the related area under the curve (AUC) to assess the ability of cQUS parameters to identify osteopenia. The presence of osteopenia was chosen as outcome variable to investigate the reliability of cQUS since in patients with acromegaly the DXA evaluation is still the gold standard for bone assessment (despite the known limitations). Vertebral fractures were not considered as an appropriate outcome variable for cQUS validation due to the low prevalence observed in our cohort. Comparison between the ROC curves was performed using the DeLong test. Furthermore, the best-fitting cQUS-derived T-score e BQI values were computed using the Youden index.

Sample size estimation was performed to determine the number of patients required to detect a significant AUC (≥ 0.8) for cQUS parameters in discriminating the presence of osteopenia defined by using DXA scan: assuming a two-sided alpha of 0.1 and using a 1:1 ratio between acromegaly patients and controls, the analysis indicated a minimum requirement of 39 subjects per group. Differences were taken to be statistically significant at *p* < 0.05.

## Results

### Patients’ characteristics

We included 56 patients with acromegaly, 25 (45%) females, median age at the time of study inclusion 59.0 years (IQR 52.0 to 64.8 years), and median BMI 26.0 kg/m^2^ (IQR 23.9 to 29.4 kg/m^2^). We compared the data of patients with acromegaly with 59 healthy controls, matched for gender (*p* = 0.908), age (*p* = 0.183), and BMI (*p* = 0.383) (see Table 1).

**Table 1:** Demographic and clinical data of patients with acromegaly and the control group

Data	Patients (*n* = 56)	Healthy controls (*n* = 59)	*p*-value
Sex (F; n,%)	25 (45%)	28 (47%)	0.908
Age (years)	59.0 (IQR 52.0-64.8)	58.0 (IQR 50.0-60.5)	0.183
BMI (kg/m^2^)	26.0 (IQR 23.9–29.4)	25.4 (IQR 23.3–29.4)	0.383
Hypogonadism/menopause (n, %)	25 (45%)	24 (41%)	0.810
Hypothyroidism (n, %)	13 (23%)^a^		
Central adrenal insufficiency (n, %)	3 (5%)		
Treatment modalities			
Neurosurgery (n,%)	40 (71%)		
Radiotherapy (n,%)	2 (4%)		
Medical therapy	41 (73%)		
Fg-SRL (n,%)	28 (53%)		
PEG (n,%)	9 (16%)		
CAB (n,%)	4 (7%)		
Pasireotide (n,%)	9 (16%)		
Biochemical values (last follow-up)			
IGF-1 absolute (µg/L)	172.0 (IQR 135.0- 203.0)		
IGF-1 xULN	0.90 (IQR 0.73–1.17)		
Biochemical control^b^ (n,%)	35 (63%)		
DXA data			
L1-L4 BMD (g/cm²)	1.14 (IQR 1.07–1.40)	1.15 (IQR 1.06–1.29)	0.447
L1-L4 T-score	-0.40 (IQR − 1.00–1.58)	-0.50 (IQR − 1.10–0.65)	0.441
TBS	1.330 (IQR 1.240–1.380)	1.410 (IQR 1.360–1.470)	*p* < 0.001
T-score modified by TBS	-1.30 (IQR − 2.12 – -0.78)	-0.40 (IQR − 1.15–0.15)	*p* < 0.001
Femoral neck BMD (g/cm²)	0.94 (IQR 0.83–1.03)	0.88 (IQR 0.79–0.98)	0.238
Femoral neck T-score	-0.80 (IQR − 1.70 − 0.03)	-1.10 (IQR − 1.70 - -0.70)	0.075
Total femoral BMD (g/cm²)	1.02 (IQR 0.90–1.10)	1.03 (IQR 0.91–1.12)	0.648
Total femoral T-score	-0.40 (IQR − 1.10–0.50)	-0.40 (IQR − 1.00–0.25)	0.871
Bone mineral metabolism markers			
PTH (ng/L)	55.0 (IQR 41.5–66.8)		
25(OH) vitamin D (ng/mL)	28.6 (IQR 6.72–31.4)		
Calcium^c^ (mg/dL)	9.24 (IQR 9.24–0.55)		
Phosphorus (mg/dL)	3.30 (IQR 3.00–3.70)		
BSAP (µg/L)	13 (IQR 10.8–16.9)		
CTX (ng/ml)	0.194 (IQR 0.080–0.280)		

**Legend: ***F* females, *SD* standard deviation, *BMI* body mass index, *fg-SRL* first-generation somatostatin receptor ligand, *PEG* pegvisomant, *CAB* cabergoline, *IGF-1* insulin-like growth factor 1, *ULN* upper limit of normality range, *PTH* parathyroid hormone, *BSAP* bone- specific alkaline phosphatase, *CTX* C-terminal telopeptide

^a^2 of the 13 patients with hypothyroidism had central hypothyroidism; ^b^Biochemical control has been defined as IGF-1 ≤ 1.0 xULN.ccalcium levels corrected for albumin levels. All continuous data are expressed as median and interquartile range

The majority of patients (*n* = 35, 63%) had a controlled disease (defined as age-adjusted IGF-1 levels ≤ 1.0 xULN) and the overall median IGF-1 xULN was 0.90 xULN (IQR 0.73 to 1.17).

Nine patients (16%) had T2DM; only two subjects had uncontrolled glycaemic control at the time of DXA scan. Considering the whole cohort of the AG, patients had a median HbA1c of 5.8% [IQR 5.6 to 6.05%] and a median plasma glucose of 96 mg/dL [IQR 89 to 104 mg/dL]).

Bone mineral metabolism and bone turnover markers were in the normal range (Table 1), except for 25(OH)D (median value 28.6 ng/mL, IQR 6.72 to 31.4 ng/mL). In particular, six (11%) patients had 25(OH)D insufficiency (i.e., 25(OH) D < 20 ng/mL [25]) and 45 (80%) patients were supplemented with cholecalciferol. Of note, four (7%) patients were treated with bone active drugs; in detail, three patients were treated with bisphosphonates and one patient with denosumab. Three patients (5%) had a history of vertebral fractures. We could not perform a statistically sound comparison of the DXA and cQUS values between patients with and without a history of vertebral fractures, due to the low number of events.

### DXA scan data

As expected, we did not find a significant difference in the BMD values and T-scores both at the lumbar and hip level between AG and CG, whereas TBS values were significantly lower in the AG compared to the CG (see Table 1).

According to the definition reported in the Method section, 5 patients (9%) of the AG had osteoporosis, 28 (50%) had osteopenia, and 23 (41%) had normal DXA values. In the CG, by definition, no subject had osteoporosis, while 41 (70%) subjects had osteopenia and 18 (30%) had normal DXA values. When using the T-score modified according to the TBS values, 12 (21%) patients of the AG had osteoporosis, 28 (50%) had osteopenia and 16 (29%) had normal DXA values; in the CG, by definition, no subject had osteoporosis, 44 (75%) subjects had osteopenia and 15 (25%) had normal DXA values. The prevalence of osteopenia in the CG was higher than that reported in age-matched samples from the general population (75% vs. 50%) [26, 27]. However, because individuals with osteoporosis, which account for approximately 20–35% of the general population, were excluded from our cohort, this may have artificially increased the proportion of osteopenia within the CG, thereby limiting the direct comparability of these estimates.

**Fig. 1:**
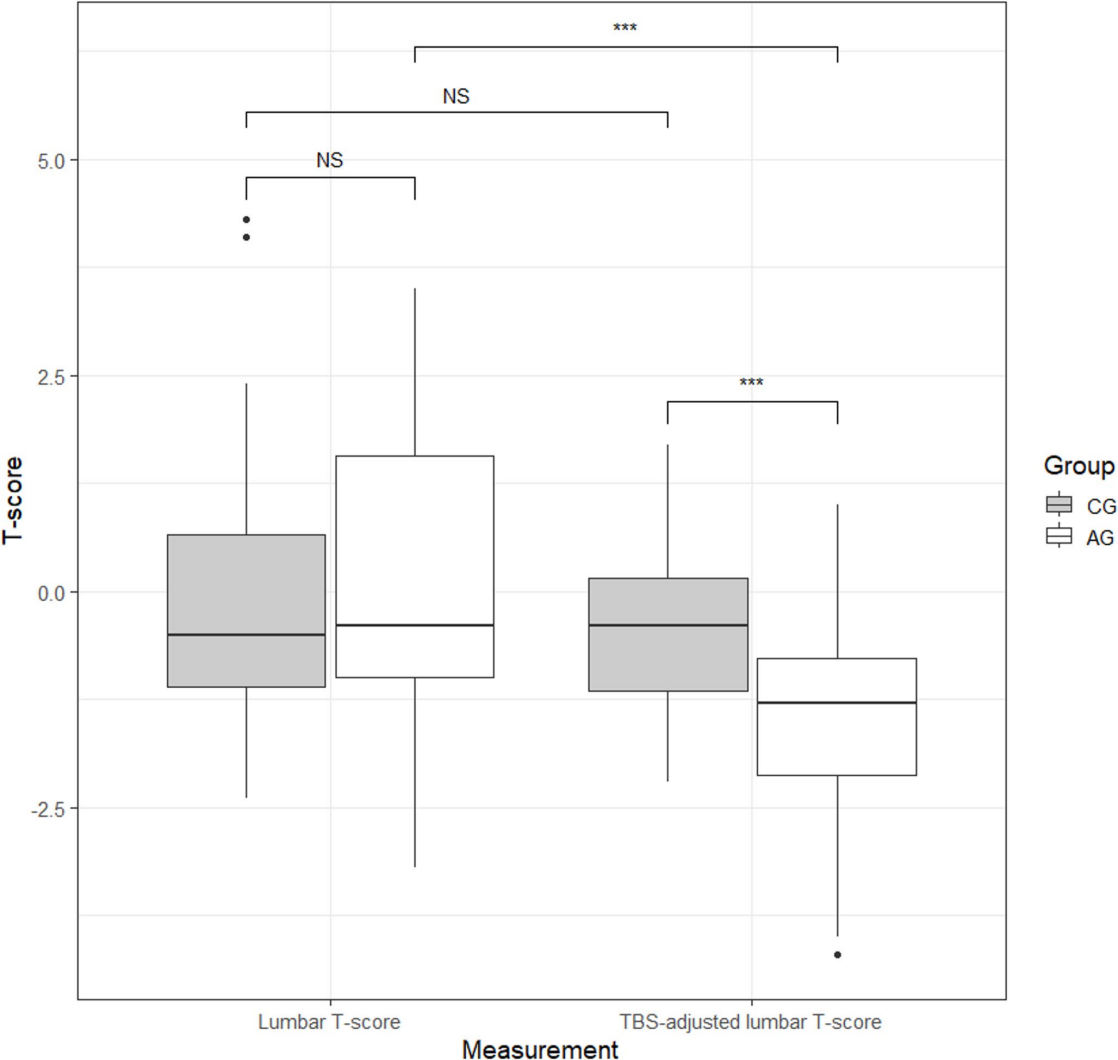
Difference in DXA-derived T-scores and TBS-adjusted DXA-derived T-scores between patients with acromegaly (acromegaly group, AG) and healthy controls (control group, CG)

As expected, the AG showed more frequently bone microarchitecture impairment compared to the CG (*p* < 0.001). In particular, nine (16%) patients with acromegaly showed degraded bone microarchitecture, 23 (41%) showed partially degraded microarchitecture and 24 (43%) had normal microarchitecture. On the other hand, within the CG no subject had degraded microarchitecture and the vast majority of subjects (*n* = 48, 81%) had normal microarchitecture. The TBS-adjusted lumbar T-score was significantly lower in the AG compared to the CG (AG: -1.30 [IQR − 2.12 to -0.78], CG: -0.40 [IQR − 1.15 to 0.15], *p* < 0.001). Of note, in the AG the TBS-adjusted lumbar T-score values were significantly lower compared with the “standard” BMD-derived T-score (*p* < 0.001), while no difference was observed in the CG between these two parameters (*p* = 0.138, Fig. 1).

Of note, repeating the analysis excluding the three patients with evidence of vertebral fractures at the morphometry examination did not modify the results (data not shown).

As previously reported, no DXA value correlated with IGF-1 xULN values, and no statistically significant difference was observed between patients with controlled disease and those with active disease. Moreover, we did not find a significant impact of pituitary deficiencies, including hypogonadism, on DXA values. All DXA values, except lumbar T-score, showed a negative correlation with BSAP (see Supplementary Table 1).

### cQUS data

All cQUS parameters showed a good correlation with the DXA-derived T-score values (lumbar, femoral neck and total hip sites) in both the AG and the CG (Table 2).

**Table 2:** Correlation data between cQUS parameters and DXA-derived T-score, TBS and TBS-adjusted T-score

	BQI		cQUS-derived T-score		SOS		BUA	
	Rho	*p*-value	Rho	*p*-value	Rho	*p*-value	Rho	*p*-value
**Acromegaly group (AG)**								
Lumbar T-score	**0.430**	< **0.001**	**0.640**	**< 0.001**	**0.540**	**< 0.001**	**0.580**	< **0.001**
TBS	0.190	0.170	0.270	0.055	0.240	0.090	0.210	0.140
TBS-adjusted lumbar T-score	0.210	0.130	**0.300**	**0.029**	**0.280**	**0.045**	0.250	0.070
Total hip T-score	**0.560**	**< 0.001**	**0.570**	**< 0.001**	**0.540**	**< 0.001**	**0.420**	**0.002**
Femoral neck T-score	**0.620**	**< 0.001**	**0.650**	**< 0.001**	**0.610**	< **0.001**	**0.490**	**< 0.001**
**Control group (CG)**								
Lumbar T-score	**0.590**	**< 0.001**	**0.570**	**< 0.001**	**0.440**	**< 0.001**	**0.310**	**0.015**
TBS	0.150	0.240	0.210	0.110	0.071	0.590	0.090	0.500
TBS-adjusted lumbar T-score	0.200	0.140	0.240	0.067	0.100	0.440	0.130	0.310
Total hip T-score	**0.630**	< **0.001**	**0.770**	**< 0.001**	**0.590**	**< 0.001**	**0.510**	**< 0.001**
Femoral neck T-score	**0.490**	< **0.001**	**0.700**	**< 0.001**	**0.560**	**< 0.001**	**0.440**	**< 0.001**

**Legend: ***cQUS* calcaneal quantitative ultrasound, *BQI* bone quality index, *SOS* speed of sound, *BUA* broadband ultrasound attenuation, *TBS* trabecular bone score. Statistically significant correlations are reported in bold

On the contrary, no correlation was observed between cQUS parameters and TBS values in both groups. No correlation was observed between lumbar T-score values adjusted by TBS and cQUS parameters in the CG; however, in the AG we observed a significant positive correlation between lumbar TBS-adjusted T-score values and both cQUS-derived T-score (rho = 0.300, *p* = 0.029) and SOS (rho = 0.280, *p* = 0.045, Table 2).

Similarly to standard DXA values, we did not find a significant difference between the AG and the CG in the BQI values (AG: 96.6 [IQR 79 to 134]; CG: 85.9 [IQR 77.1 to 103], *p* = 0.091), as well as in the cQUS-derived T-score (AG: 0.40 [IQR − 1.60 to 1.50]; CG: -1,30 [IQR − 1.65 to 0.95], *p* = 0.068), although numerically higher values were observed in the AG.

**Fig. 2:**
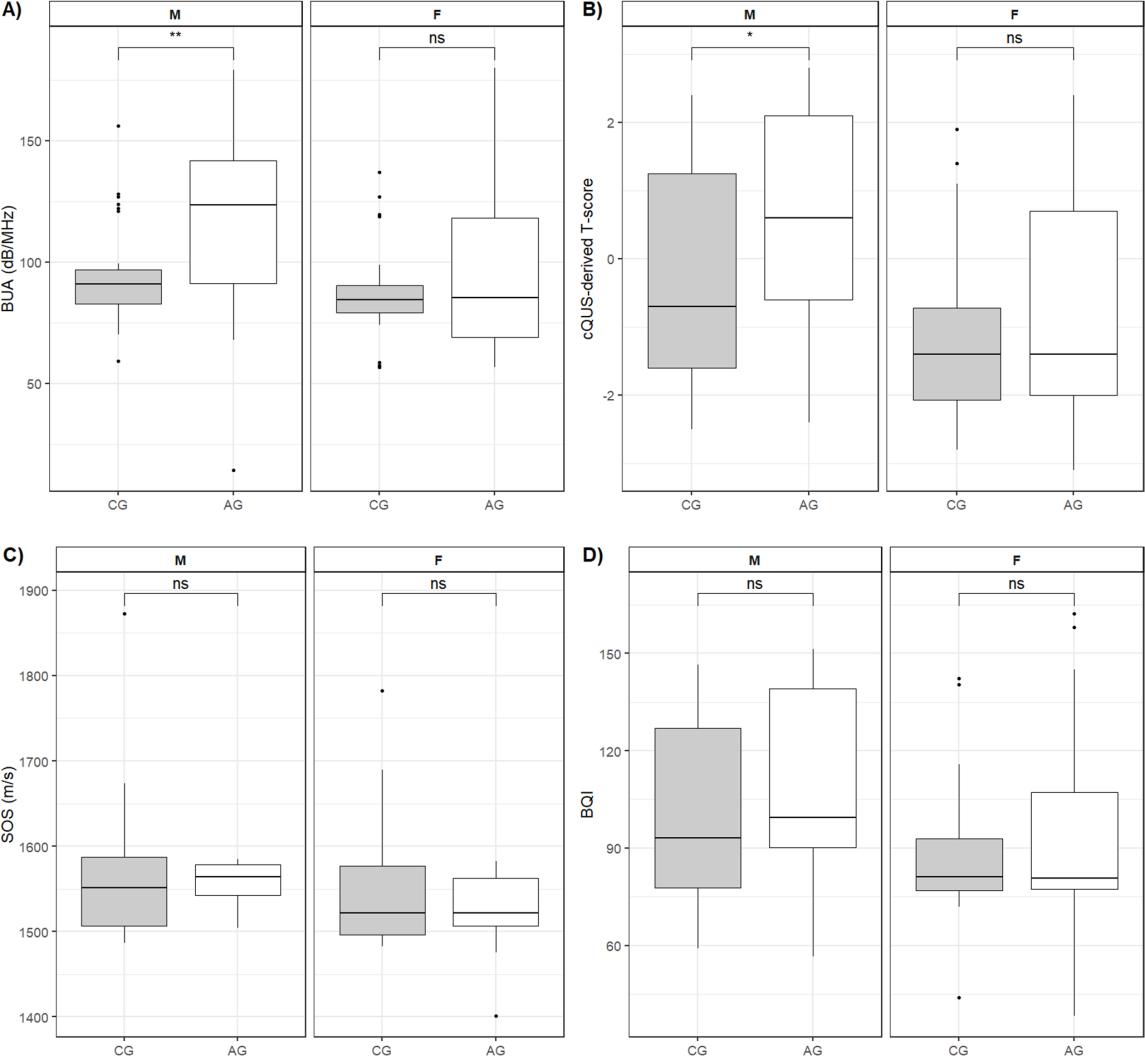
Difference in cQUS parameters between patients with acromegaly (AG) and healthy controls (CG), stratified by sex: panel A, BUA; panel B, cQUS-derived T-score; panel C, SOS; panel D, BQI. Legend: cQUS, calcaneal quantitative ultrasound; BQI, bone quality index; SOS, speed of sound; BUA, broadband ultrasound attenuation

When data were stratified based on sex, male patients in the AG (0.60 [IQR − 0.60 to 2.10]) showed significantly higher cQUS-derived T-score values compared to the CG subjects (-0.70 [IQR − 1.60 to 1.25], *p* = 0.040) (Fig. 2B). On the other hand, no significant differences were observed for BQI values and cQUS-derived T-score between the AG and the CG in female subjects (Fig. 2A-B).

We then analysed the two components determining the cQUS BQI (namely, SOS and BUA). We did not observe statistically significant differences in SOS values between the AG (1560 m/s [IQR 1541 to 1575 m/s]) and the CG (1531 m/s [IQR 1501 to 1582 m/s], *p* = 0.790), irrespective of subjects’ sex (Fig. 2C). As concern the BUA, we observed significantly higher values in the AG (112.0 dB/MHz [IQR 77.6 to 126.0 dB/MHz]) compared to the CG (89.7 dB/MHz [IQR 79.2 to 93.1], *p* = 0.036). When stratifying by sex, this difference was statistically significant only in male subjects (AG: 123.6 dB/MHz [IQR 91.10 to 141.7] vs. CG: 92.8 dB/MHz [IQR 82.5 to 96.95], *p* = 0.005), whereas in female subjects the median values were almost superimposable (AG: 85.3 dB/MHz [IQR 68.9 to 118.1] vs. CG: 84.4 dB/MHz [IQR 79.2 to 90.4], *p* = 0.970; Fig. 2D).

In the AG, no cQUS parameter correlated with IGF-1 xULN values, and no statistically significant differences were observed in cQUS parameters between patients who achieved disease control and patients with active disease. Moreover, we did not find a significant impact of pituitary deficiencies, including hypogonadism, on cQUS parameters. As concerns the specific bone markers, only BSAP showed a negative correlation with all cQUS parameters except cQUS-derived T-score, that showed a trend not reaching statistical significance (Supplementary Table 1).

### Predictive value of cQUS on DXA parameters

We included in multivariable linear regression analyses all variables of clinical interest and those that showed statistically significant correlations with DXA values: age, sex, presence of hypogonadism, DM, controlled acromegaly and BSAP values. Among the cQUS parameters, to optimize the number of variables in the model, we chose to include in the multivariable linear regression analyses the cQUS-derived T-score, as it showed the best overall performance (see Table 2).

In the AG, the final model to identify predictors of lumbar T-score retained cQUS-derived T-score and disease control, explaining about 40% of lumbar T-score variance (R^2^: 0396, adjusted R^2^: 0.348, *p* = 0.002; Table 3).

**Table 3:** Multivariable logistic regression to determine predictors of TBS values and DXA-derived T-score (at different sites) in patients with acromegaly

Dependent variables	Independent variables	β (estimate)	95% CI	*p*-value
Lumbar T-score	QUS T-score	0.688	0.327 to 1.050	**< 0.001**
	Active acromegaly	-0.933	-2.240 to 0.374	0.154
Femoral neck T-score	QUS T-score	0.313	0.102 to 0.524	**0.005**
	Age	-0.069	-0.103 to -0.035	**< 0.001**
	Active acromegaly	-0.555	-1.250 to 0.136	0.110
Total hip T-score	QUS T-score	0.329	0.127 to 0.530	**0.003**
	Age	-0.058	-0.089 to -0.027	**< 0.001**
	Sex (F)	-0.402	-1.010 to 0.207	0.186

**Legend: ***cQUS* calcaneal quantitative ultrasound. Statistically significant correlations are reported in bold

As concern femoral neck T-score, the model retained cQUS-derived T-score, patients’ age and disease control, explaining 66% of the observed variance (R^2^: 0.668, adjusted R^2^: 0.626, *p* < 0.001; Table 3); similarly, for the total hip T-score, the model retained cQUS-derived T-score, patients’ age and sex, thus explaining 69% of variance (R^2^: 0.687, adjusted R^2^: 0.648, *p* < 0.001).

**Fig. 3:**
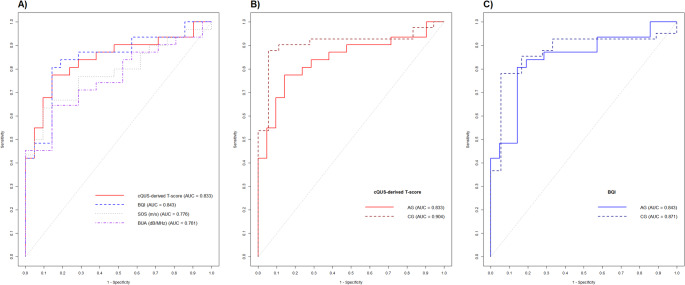
ROC curve showing the discriminatory ability of cQUS parameter to identify osteopenia in patients with acromegaly (Panel **A**); ROC curve showing the discriminatory ability of cQUS-derived T-score (panel **B**) and BQI (Panel **C**) to identify osteopenia in patients with acromegaly (acromegaly group, AG) and healthy controls (control group, CG). Legend: ROC, receiver operating characteristic; cQUS, calcaneal quantitative ultrasound; BQI, bone quality index

Finally, we evaluated the ability of cQUS parameters to identify individuals with osteopenia, classified according to DXA values, as detailed in the Method section. In the AG, BQI values and the cQUS-derived T-score showed good discriminatory ability for the presence of osteopenia (BQI: AUC 0.843 [CI 0.732–0.953]; cQUS-derived T-score: AUC 0.833 [CI 0.721–0.945]), whereas SOS (AUC 0.776 [CI 0.649–0.904]) and BUA (AUC 0.761 [CI 0.632–0.891]) values showed only an acceptable discriminatory ability (Fig. 3A). The discriminatory ability for the presence of osteopenia of cQUS-derived T-score and BQI was not significantly different (*p* = 0.754).

In the CG, all cQUS parameters, except the BUA, showed a good discriminatory ability for the presence of osteopenia. In detail, BQI had an AUC of 0.871 (CI 0.772–0.971), cQUS-derived T-score 0.904 (CI 0.818–0.990), SOS 0.828 (CI 0.723–0.933). The BUA had an AUC of 0.730 (CI 0.570–0.891).

Of note, the discriminatory ability of the cQUS-derived T-score and BQI in predicting the presence of osteopenia was not significantly different between AG and CG (*p* = 0.325 and *p* = 0.706, respectively, Fig. 3B and C).

The best cut-off to maximize accuracy in the AG was a cQUS-derived T-score of -0.15, with a sensitivity of 68% and a specificity of 90%.

Similarly, when evaluating the BQI, the best cut-off to maximize accuracy in the AG was a BQI of 97.8, with a sensitivity of 81% and a specificity of 86%.

As expected, the performance of cQUS parameters was not satisfactory to predict a degraded TBS (AUC between 0.569 and 0.646) or a partially degraded TBS score (AUC between 0.476 and 0.594) in both AG and CG.

## Discussion

We investigated the relationship between cQUS parameters and DXA measurements in a cohort of patients with acromegaly. cQUS has been shown to be an easy-to-use, radiation-free, and affordable method in various conditions, when compared to standard DXA evaluation [9–12, 28].

To date, a standard DXA scan every 2 years, and a vertebral morphometry, is recommended to evaluate the bone status in patients with acromegaly [3, 29].

In our study we highlight that the cQUS evaluation has a strong correlation with DXA-derived T-scores, with a good performance in both the AG and the CG. In detail, we found no difference between the AG and the CG in all cQUS parameters except for BUA, which resulted significantly higher in patients with acromegaly compared to the healthy controls. Of note, after stratification based on sex, only male patients with acromegaly showed significantly higher cQUS-derived T-score values and BUA values compared to male healthy controls. Two previous studies evaluated the role of cQUS in patients with acromegaly, reporting conflicting results. Bolanowski and colleagues did not find any difference between patients with acromegaly and healthy controls, irrespective of the cQUS parameter investigated, while Kastelan and colleagues found lower SOS, BUA and BQI values in male patients with acromegaly compared to controls [13, 14]. The discrepancy with our findings could be due to multiple factors. First, our CG showed a higher prevalence of subjects with osteopenia compared to available epidemiological data in the general population. This could have affected our ability to detect small differences between the two groups. Then, most of the patients included in our cohort had a controlled disease (63%, overall median IGF-1 0.90 xULN), while in the previous studies most patients had an active disease. Disease activity may impact cQUS measurements, as persistently elevated GH levels cause sodium retention and therefore soft tissue swelling [30, 31]. In particular, subcutaneous oedema, as well as increased skin thickness, could lower both SOS and BUA measurements, and therefore BQI and cQUS-derived T-score, leading to false positive findings (i.e. evidence for impaired bone status) [32, 33].

On the other hand, the higher values of cQUS-derived T-score and BUA we observed in male patients with acromegaly compared to healthy subjects could be due to the presence of osteoarthritis in patients with acromegaly that could manifest with degenerative changes as well as the development of osteophytes [34]. In line with previous studies evaluating DXA outcome in patients with acromegaly, we did not observe a correlation between disease activity and DXA parameters [8, 16]. Similarly, we did not find any correlation between disease activity and cQUS parameters.

Noteworthy, the data from the previous reports investigating the role of cQUS in patients with acromegaly cannot be directly compared with the present study, due to the use of different devices [35]. The Osteosys BeeTLE, used in the current study, has been recently shown to have a strong correlation with DXA-derived BMD and a promising diagnostic accuracy in the general population [24].

Finally, we investigated the ability of cQUS, particularly the cQUS-derived T-score, to detect osteopenia in patients with acromegaly. In our cohort, the cQUS derived T-score was a significant predictor of DXA-derived T-score and showed a good discriminatory ability to identify patients with osteopenia. The performance we observed in our patients was superimposable to the one reported in the general population and in patients with other diseases than acromegaly [10, 12, 24].

Our study has some limitations. First, as previously mentioned, the prevalence of osteopenia in the CG was higher than expected. Since we excluded subjects with osteoporosis from the CG, we could have artificially increased the prevalence of osteopenia in this group. Nevertheless, the high prevalence of mild bone impairment in the CG could have impacted our ability of detecting differences in bone health parameters between the two groups (i.e. CG vs. AG). Second, the relatively small sample size did not allow us to perform a sub-analysis based on patients’ treatment or to evaluate the fracture risk. In particular, in our cohort only three out of 19 patients who performed vertebral morphometry, had evidence of vertebral fracture and therefore we could not assess the discriminatory ability of cQUS in identifying patients at risk of vertebral fracture. However, a recent longitudinal study performed in a large cohort of post-menopausal women, showed that cQUS could predict fractures independently of DXA-derived BMD, TBS and FRAX [9]. Therefore, further studies, with a larger sample size and a control group with a lower prevalence of osteopenia, are needed to evaluate this aspect in patients with acromegaly.

In conclusion, we demonstrated that cQUS parameters showed a good correlation with all conventional DXA parameters in patients with acromegaly. Furthermore, cQUS-derived T-score was able to discriminate osteopenia with a good accuracy in a cohort of patients with mildly active/controlled acromegaly.

## Supplementary Information

Below is the link to the supplementary material.

Supplementary Material 1

## Data Availability

The datasets generated and/or analyzed during the current study are available from the corresponding author on reasonable request.

## Abbreviations

AUC: Area Under the Curve
BMD: Bone Mineral Density
BSAP: Bone Specific Alkaline Phosphatase
BUA: Broadband Ultrasound Attenuation
CTX: C-terminal telopeptide
cQUS: calcaneal Quantitative Ultrasound
DXA: Dual-energy X-ray Absorptiometry
DM: Diabetes Mellitus
fT4: free Thyroxine
GH: Growth Hormone
HbA1c: Glycated Haemoglobin
IGF-1: Insulin-like Growth Factor 1
PTH: Parathyroid Hormone
ROC: Receiver Operating Characteristic
SOS: Speed of Sound
TSH: Thyroid Stimulating Hormone
TBS: Trabecular Bone Score
ULN: Upper Limit of Normality
25(OH)D: 25-hydroxyvitamin D

## References

[1] Fleseriu M, Langlois F, Lim DST, Varlamov EV, Melmed S (2022) Acromegaly: pathogenesis, diagnosis, and management. Lancet Diabetes Endocrinol 10:804–826. 10.1016/S2213-8587(22)00244-3 36209758 10.1016/S2213-8587(22)00244-3

[2] Giustina A, Colao A, Acromegaly (2025) N Engl J Med Mass Med Soc 393:1926–1939. 10.1056/NEJMra2409076 10.1056/NEJMra2409076 41223366

[3] Giustina A, Barkan A, Beckers A, Biermasz N, Biller BMK, Boguszewski C et al (2020) A consensus on the diagnosis and treatment of acromegaly comorbidities: an update. J Clin Endocrinol Metab 105:dgz096. 10.1210/clinem/dgz096 31606735 10.1210/clinem/dgz096

[4] Frara S, Acanfora M, Franzese V, Brandi ML, Losa M, Giustina A (2024) Novel approach to bone comorbidity in resistant acromegaly. Pituitary 27:813–823. 10.1007/s11102-024-01468-y 39570564 10.1007/s11102-024-01468-y

[5] Chiloiro S, Palumbo C, Giampietro A, De Marinis L, Bianchi A, Giustina A et al (2025) Acromegaly treatment and bone: a bidirectional relationship. Pituitary 28:124. 10.1007/s11102-025-01573-6 41205005 10.1007/s11102-025-01573-6 PMC12596337

[6] Sorohan MC, Poiana C (2023) Vertebral fractures in acromegaly: a systematic review. J Clin Med Multidisciplinary Digit Publishing Inst 12:164. 10.3390/jcm12010164 10.3390/jcm12010164 PMC9821150 36614962

[7] Mazziotti G, Gola M, Bianchi A, Porcelli T, Giampietro A, Cimino V et al (2011) Influence of diabetes mellitus on vertebral fractures in men with acromegaly. Endocrine 40:102–108. 10.1007/s12020-011-9486-x 21594681 10.1007/s12020-011-9486-x

[8] Bioletto F, Barale M, Prencipe N, Berton AM, Parasiliti-Caprino M, Gasco V et al (2023) Trabecular bone score as an index of bone fragility in patients with acromegaly: a systematic review and meta-analysis. Neuroendocrinology 113:395–405. 10.1159/000528199 36617407 10.1159/000528199

[9] Métrailler A, Hans D, Lamy O, Gonzalez Rodriguez E, Shevroja E (2023) Heel quantitative ultrasound (QUS) predicts incident fractures independently of trabecular bone score (TBS), bone mineral density (BMD), and FRAX: the OsteoLaus Study. Osteoporos Int 34:1401–1409. 10.1007/s00198-023-06728-4 37154943 10.1007/s00198-023-06728-4 PMC10382398

[10] Nieuwkamer BB, Vrouwe JPM, Willemse PM, Nicolai MPJ, Bevers RFM, Pelger RCM et al (2023) Quantitative ultrasound of the calcaneus (QUS): a valuable tool in the identification of patients with non-metastatic prostate cancer requiring screening for osteoporosis. Bone Rep 18:101679. 10.1016/j.bonr.2023.101679 37425192 10.1016/j.bonr.2023.101679 PMC10323220

[11] Khandelwal N, Rajauria S, Kanjalkar SP, Chavanke OS, Rai S, Khandelwal N et al (2023) Bone mineral density evaluation among type 2 diabetic patients in rural Haryana, India: an analytical cross-sectional study. Cureus [Internet] Cureus. [cited 2025 June 23];15 10.7759/cureus.45908 37885541 10.7759/cureus.45908 PMC10599097

[12] Chanprasertpinyo W, Punsawad C, Khwanchuea R, Sukkriang N, Yincharoen P, Rerkswattavorn C (2023) Comparison between calcaneus quantitative ultrasound and the gold standard DXA in the ability to detect osteoporosis in chronic obstructive pulmonary disease patients. J Orthop Surg 18:778. 10.1186/s13018-023-04211-8 10.1186/s13018-023-04211-8 PMC10577968 37845656

[13] Bolanowski M, Jędrzejuk D, Milewicz A, Arkowska A (2002) Quantitative ultrasound of the heel and some parameters of bone turnover in patients with acromegaly. Osteoporos Int 13:303–308. 10.1007/s001980200030 12030545 10.1007/s001980200030

[14] Kastelan D, Dusek T, Kraljevic I, Polasek O, Perkovic Z, Kardum I et al (2007) Bone properties in patients with acromegaly: quantitative ultrasound of the heel. J Clin Densitom 10:327–331. 10.1016/j.jocd.2007.03.103 17543559 10.1016/j.jocd.2007.03.103

[15] Giustina A, Biermasz N, Casanueva FF, Fleseriu M, Mortini P, Strasburger C et al (2024) Consensus on criteria for acromegaly diagnosis and remission. Pituitary 27:7–22. 10.1007/s11102-023-01360-1 37923946 10.1007/s11102-023-01360-1 PMC10837217

[16] Nazzari E, Casabella A, Paolino S, Campana C, Corica G, Nista F et al (2022) Trabecular bone score as a reliable measure of lumbar spine bone microarchitecture in acromegalic patients. J Clin Med 11:6374. 10.3390/jcm11216374 36362602 10.3390/jcm11216374 PMC9656167

[17] Bhasin S, Brito JP, Cunningham GR, Hayes FJ, Hodis HN, Matsumoto AM et al (2018) Testosterone therapy in men with hypogonadism: an endocrine society* clinical practice guideline. J Clin Endocrinol Metab 103:1715–1744. 10.1210/jc.2018-00229 29562364 10.1210/jc.2018-00229

[18] Campana C, Cocchiara F, Corica G, Nista F, Arvigo M, Amarù J et al (2021) Discordant GH and IGF-1 results in treated acromegaly: impact of GH cutoffs and mean values assessment. J Clin Endocrinol Metab 106:789–801. 10.1210/clinem/dgaa859 33236108 10.1210/clinem/dgaa859

[19] Sözen T, Özışık L, Başaran NÇ (2017) An overview and management of osteoporosis. Eur J Rheumatol 4:46–56. 10.5152/eurjrheum.2016.048 28293453 10.5152/eurjrheum.2016.048 PMC5335887

[20] Silva BC, Leslie WD, Resch H, Lamy O, Lesnyak O, Binkley N et al (2014) Trabecular bone score: a noninvasive analytical method based upon the DXA image. J Bone Min Res 29:518–530. 10.1002/jbmr.2176 10.1002/jbmr.2176 24443324

[21] Leslie WD, Shevroja E, Johansson H, McCloskey EV, Harvey NC, Kanis JA et al (2018) Risk-equivalent T-score adjustment for using lumbar spine trabecular bone score (TBS): the manitoba BMD registry. Osteoporos Int J Establ Result Coop Eur Found Osteoporos Natl Osteoporos Found USA 29:751–758. 10.1007/s00198-018-4405-0 10.1007/s00198-018-4405-0 PMC5993188 29392355

[22] Wear KA, Nagaraja S, Dreher ML, Sadoughi S, Zhu S, Keaveny TM (2017) Relationships among ultrasonic and mechanical properties of cancellous bone in human calcaneus in vitro. Bone 103:93–101. 10.1016/j.bone.2017.06.021 28666970 10.1016/j.bone.2017.06.021 PMC6941483

[23] Bouxsein ML, Radloff SE (1997) Quantitative ultrasound of the calcaneus reflects the mechanical properties of calcaneal trabecular bone. J Bone Min Res Off J Am Soc Bone Min Res 12:839–846. 10.1359/jbmr.1997.12.5.839 10.1359/jbmr.1997.12.5.839 9144351

[24] Adami G, Rossini M, Gatti D, Serpi P, Fabrizio C, Lovato R (2024) New point-of-care calcaneal ultrasound densitometer (Osteosys BeeTLE) compared to standard dual-energy X-ray absorptiometry (DXA). Sci Rep Nat Publishing Group 14:6898. 10.1038/s41598-024-56787-8 10.1038/s41598-024-56787-8 PMC10959987 38519548

[25] Giustina A, Bilezikian JP, Adler RA, Banfi G, Bikle DD, Binkley NC et al (2024) Consensus statement on vitamin D status assessment and supplementation: Whys, whens, and hows. Endocr Rev 45:625–654. 10.1210/endrev/bnae009 38676447 10.1210/endrev/bnae009 PMC11405507

[26] Cipriani C, Pepe J, Bertoldo F, Bianchi G, Cantatore FP, Corrado A et al (2018) The epidemiology of osteoporosis in Italian postmenopausal women according to the National Bone Health Alliance (NBHA) diagnostic criteria: a multicenter cohort study. J Endocrinol Invest 41:431–438. 10.1007/s40618-017-0761-4 28956296 10.1007/s40618-017-0761-4

[27] Sarafrazi N, Wambogo EA, Shepherd JA (2021) Osteoporosis or low bone mass in older adults: United States, 2017–2018. NCHS Data Brief 1–8 34029181

[28] Thomsen K, Jepsen DB, Matzen L, Hermann AP, Masud T, Ryg J (2015) Is calcaneal quantitative ultrasound useful as a prescreen stratification tool for osteoporosis? Osteoporos Int 26:1459–1475. 10.1007/s00198-014-3012-y 25634771 10.1007/s00198-014-3012-y

[29] Giustina A (2023) Acromegaly and Bone: An Update. Endocrinol Metab Korean Endocr Soc 38:655–666. 10.3803/EnM.2023.601 10.3803/EnM.2023.601 PMC10764988 38164073

[30] Kamenicky P, Blanchard A, Frank M, Salenave S, Letierce A, Azizi M et al (2011) Body fluid expansion in acromegaly is related to enhanced epithelial sodium channel (ENaC) activity. J Clin Endocrinol Metab 96:2127–2135. 10.1210/jc.2011-0078 21508131 10.1210/jc.2011-0078

[31] Varaldo E, Prencipe N, Berton AM, Cuboni D, Aversa LS, Sibilla M et al (2025) Evaluation of fluid status in patients with acromegaly through bioelectrical impedance vector analysis: a cross-sectional study. J Endocrinol Invest 48:1185–1195. 10.1007/s40618-025-02541-4 39954196 10.1007/s40618-025-02541-4 PMC12049396

[32] Johansen A, Stone MD (1997) The effect of ankle oedema on bone ultrasound assessment at the heel. Osteoporos Int 7:44–47. 10.1007/BF01623459 9102062 10.1007/BF01623459

[33] Chappard C, Camus E, Lefebvre F, Guillot G, Bittoun J, Berger G et al (2000) Evaluation of error bounds on calcaneal speed of sound caused by surrounding soft tissue. J Clin Densitom Off J Int Soc Clin Densitom 3:121–131. 10.1385/jcd:3:2:121 10.1385/jcd:3:2:121 10871906

[34] Killinger Z, Kužma M, Sterančáková L, Payer J (2012) Osteoarticular Changes in Acromegaly. Int J Endocrinol 2012:839282. 10.1155/2012/839282 23008710 10.1155/2012/839282 PMC3447355

[35] Hans D, Métrailler A, Gonzalez Rodriguez E, Lamy O, Shevroja E (2022) Quantitative Ultrasound (QUS) in the Management of Osteoporosis and Assessment of Fracture Risk: An Update. In: Laugier P, Grimal Q (eds) Bone Quant Ultrasound New Horiz [Internet]. Springer International Publishing, Cham, pp 7–34. [cited 2025 Aug 27] 10.1007/978-3-030-91979-5_2 10.1007/978-3-030-91979-5_2 35508869

