# Supplementary material for: Calcaneal quantitative ultrasound parameters strongly correlate with DXA measurements in patients with acromegaly
Source: Pituitary. 2026 May 18;29(3):86. doi: 10.1007/s11102-026-01696-4 (PMC13183734; doi:10.1007/s11102-026-01696-4)
Supplement: Supplementary file 1 — Supplementary Material 1 [file 11102_2026_1696_MOESM1_ESM.docx]

**Supplementary Table 1.** Correlation data between bone osteometabolic markers, cQUS and DXA-derived parameters in patients with acromegaly (acromegaly group, AG).

|  | BQI | | cQUS-derived  T-score | | SOS | | BUA | |
| --- | --- | --- | --- | --- | --- | --- | --- | --- |
|  | Rho | p-value | Rho | p-value | Rho | p-value | Rho | p-value |
| PTH (ng/L) | -0.200 | 0.229 | -0.283 | 0.085 | **-0.341** | **0.036** | **-0.443** | **0.005** |
| 25(OH) vitamin D (ng/mL) | 0.084 | 0.581 | 0.110 | 0.468 | 0.153 | 0.311 | 0.219 | 0.144 |
| Calcium* (mg/dL) | -0.086 | 0.557 | -0.149 | 0.308 | -0.115 | 0.430 | -0.059 | 0.685 |
| Phosphorus (mg/dL) | -0.108 | 0.535 | -0.064 | 0.713 | -0.149 | 0.394 | -0.032 | 0.854 |
| BSAP (µg/L) | **-0.460** | **0.011** | -0.360 | 0.053 | **-0.600** | **<0.001** | **-0.440** | **0.015** |
| CTX (ng/ml) | -0.291 | 0.126 | -0.188 | 0.329 | **-0.380** | **0.043** | -0.197 | 0.306 |

|  |  |  |  |  |  |  |
| --- | --- | --- | --- | --- | --- | --- |
|  | **Lumbar T-score** | | **Total hip T-score** | | **Femoral neck T-score** | |
|  | Rho | p-value | Rho | p-value | Rho | p-value |
| PTH (ng/L) | -0.133 | 0.412 | -0.137 | 0.398 | -0.075 | 0.647 |
| 25(OH) vitamin D (ng/mL) | 0.001 | 0.998 | -0.112 | 0.450 | -0.091 | 0.539 |
| Calcium* (mg/dL) | -0.091 | 0.518 | **-0.289** | **0.038** | -0.207 | 0.141 |
| Phosphorus (mg/dL) | -0.206 | 0.208 | -0.247 | 0.129 | -0.018 | 0.913 |
| BSAP (µg/L) | -0.320 | 0.078 | **-0.560** | **0.001** | **-0.480** | **0.006** |
| CTX (ng/ml) | **-0.370** | **0.043** | **-0.400** | **0.027** | -0.319 | 0.086 |
|  |  |  |  |  |  |  |
|  | **TBS** | | **TBS-adjusted lumbar T-score** | |  |  |
|  | Rho | p-value | Rho | p-value |  |  |
| PTH (ng/L) | 0.032 | 0.847 | 0.026 | 0.875 |  |  |
| 25(OH) vitamin D (ng/mL) | 0.031 | 0.833 | 0.011 | 0.943 |  |  |
| Calcium* (mg/dL) | -0.218 | 0.120 | -0.214 | 0.128 |  |  |
| Phosphorus (mg/dL) | -0.076 | 0.644 | -0.166 | 0.313 |  |  |
| BSAP (µg/L) | **-0.440** | **0.014** | **-0.400** | **0.025** |  |  |
| CTX (ng/ml) | -0.280 | 0.130 | -0.320 | 0.085 |  |  |

Legend: PTH, parathyroid hormone; BSAP, bone- specific alkaline phosphatase; CTX, C-terminal telopeptide. *Calcium corrected by albumin values. Statistically significant correlations are reported in bold.
